# 83551 Current implementation of expedited partner therapy for the treatment of N. Gonorrhoeae and C. Trachomatis infection: Integrating mixed methods with cost-effectiveness analysis

**DOI:** 10.1017/cts.2021.701

**Published:** 2021-03-30

**Authors:** Emily Ann Groene Faherty, Kumi Smith, Christy M. Boraas, Sarah M. Lofgren, Eva A. Enns

**Affiliations:** 1 University of Minnesota Clinical and Translational Science Institute; 2 University of Minnesota School of Public Health; 3 University of Minnesota Medical School

## Abstract

ABSTRACT IMPACT: This work will estimate current EPT implementation in Minnesota and provide cost-effectiveness analyses of different implementation scenarios to inform STI treatment policy. OBJECTIVES/GOALS: This research aims to 1) assess current implementation of Expedited Partner Therapy (EPT) as treatment for C. Trachomatis (chlamydia) and N. Gonorrhoeae (gonorrhea) among healthcare providers in Minnesota and to 2) simulate the current burden of chlamydia and gonorrhea infections to test the cost-effectiveness of increasing EPT implementation. METHODS/STUDY POPULATION: We will conduct key informant interviews (KII) and an online survey of health providers across the continuum of care for chlamydia and gonorrhea treatment. Based on experience in prior studies, the KII sample size is expected to be about 15 informants. KIIs will be carried out among providers who submitted EPT protocols to the Minnesota Department of Health to understand how EPT is currently being implemented. KII results will inform the online survey of health providers, which will estimate how many providers across the state provide EPT. We will distribute the survey through Minnesota health provider networks to achieve a sample of at least 500 health providers. The KII and survey results will inform model structure and parameter values for a compartmental cost-effectiveness model of EPT. RESULTS/ANTICIPATED RESULTS: Initial results from KII pilots suggest that EPT is primarily provided through a paper script for the sexual partner of a patient who tests positive for CT or NG by the treating provider. Less commonly, a patient’s partner who is already a patient in the health system may receive notification and treatment through the provider. While EPT is legal in Minnesota, concerns about medical liability for adverse reactions and difficulty obtaining paper scripts in electronic workflows are barriers to implementation. The statewide survey will include questions to estimate the likelihood of EPT provision among providers when these concerns are addressed. These figures will be integrated into the cost-effectiveness model to simulate outcomes and costs across different EPT implementation scenarios. DISCUSSION/SIGNIFICANCE OF FINDINGS: The statewide survey will define cost-effectiveness model parameters, including the proportion of providers in the state who currently provide EPT or would be willing to provide EPT under different scenarios. Study findings will be shared with health provider networks and health departments to inform STI treatment procedures and state EPT policies.

